# Health and Habit

**Published:** 1882-04

**Authors:** 


MONTHLY.

E. H. GIBBS, A. M., M. D., EDITOR.

FOUNDED IN 1854.

### HEALTH AND HABIT.

GOOD health depends more upon the habits of life of each individual than is generally supposed.

We often hear persons assert that they cannot afford the time and labor required to be systematic in their methods of living; when in fact they can't afford to do otherwise. "Order is Heaven's first law." It is method by which time is economized, labor saved and health preserved. It is the orderly persen who lives to a purpose; it is the vacillating individual who leaves the world no better for having lived in it. The most important lesson of daily practical life, to be taught to every child, is order. It is the best guarantee not only of success in the world but of health, happiness and long life. We are creatures of habit, and whatever routine we are accustomed to in youth becomes a second nature. Few parents realize their responsibility to its fullest extent. They are often responsible for many of the evils that come upon their children in later life, both as to health and morals. There are no influences so potent and enduring as those of a comfortable home where order reigns; where there is neither jar nor discord to mar its harmony; where the government is not harsh and exacting; but of the kind "that governs least."

During a long residence in France I saw nothing so beautiful as the relations almost always existing between parents and their children. They are all children together; in complete sympathy in their joys and sorrows, as in their confidences. The mistakes and follies of youth are not corrected by harsh means—they never could be effectually—but by the kindly persuasion of the parents, who seem like elder brothers and sisters. The basis of this happy condition of affairs is love for each other; and the next greatest factor is the order and decorum that maintain it. Their habits of life are like clock-work; hence, as a nation, the French people are the most sober, thrifty and industrious as well as the happiest in the world.

